# Anterior cruciate ligament reconstruction complicated by pyoderma gangrenosum

**DOI:** 10.1308/003588412X13373405387573

**Published:** 2012-05

**Authors:** E Bagouri, Jon Smith, G Geutjens

**Affiliations:** Derby Hospitals NHS Foundation Trust,UK

**Keywords:** Pyoderma gangrenosum, Anterior cruciate ligament, Cruciate

## Abstract

We report a case of pyoderma gangrenosum as a complication of an anterior cruciate ligament reconstruction in a patient with inflammatory bowel disease, which was misdiagnosed initially as a post-operative wound infection. An early dermatology opinion and skin biopsy should be considered in cases of suspected infection where thorough surgical debridement and antimicrobial therapy has failed to improve the clinical picture.

Pyoderma gangrenosum (PG) is a rare, immunological, ulcerative condition of the skin that may occur spontaneously or secondarily to injury or surgery. This condition is known to be associated with inflammatory bowel disease and, in the context of surgery, may be misdiagnosed as a post-operative wound infection. We present a case of PG as a complication of an anterior cruciate ligament (ACL) reconstruction, mimicking a surgical site infection.

## Case history

A 33-year-old male engineer presented with symptoms of right knee instability following a severe valgus twisting injury when he fell downstairs. He was known to have a history of ulcerative colitis. Following magnetic resonance imaging, he went on to have an uneventful, arthroscopically assisted ACL reconstruction using an ipsilateral hamstrings graft, with concomitant medial and lateral meniscal repairs.

Four days following surgery, the patient was admitted under the gastroenterologists with abdominal pain secondary to an acute exacerbation of his ulcerative colitis. His temperature on admission was 39°C and inflammatory markers showed a C-reactive protein (CRP) level of 329mg/l (105mg/l pre-operatively) and a white cell count of 16 × 10^9^/l. Our physicians commenced him on intravenous (IV) co-amoxiclav. At this stage, the right knee was quiescent and the post-operative wounds looked healthy.

**Figure 1:**
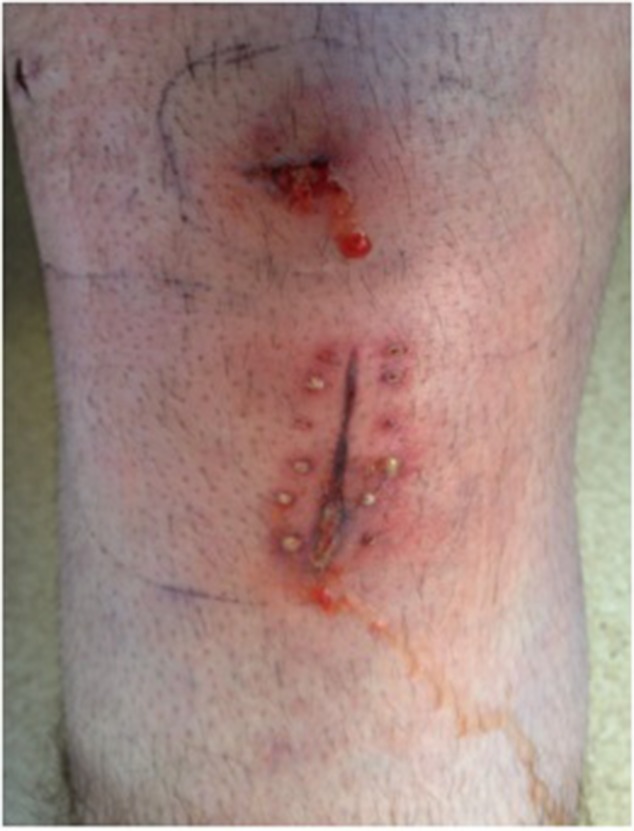
Initial appearance of wound on post-operative day 11

**Figure 2:**
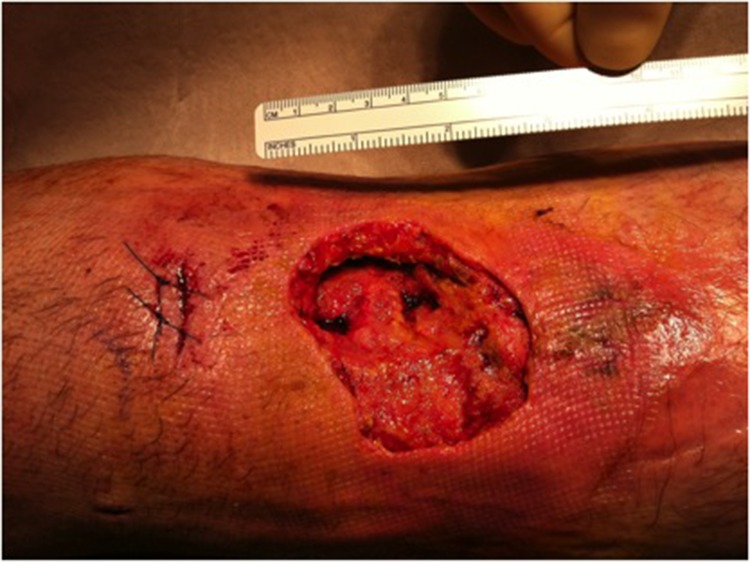
Wound following initial surgical debridement

On the tenth day after surgery, the patient had persistent fever and rigours, and the CRP level remained high (340mg/l). The IV co-amoxiclav was switched to vancomycin. The following day, the graft harvest site appeared clinically infected with significant erythema and pus-like discharge (Fig 1). Given these findings, he was taken to theatre where he underwent wound debridement and lavage with aspiration of the knee joint using a superolateral approach (Fig 2). A VAC® dressing (Kinetic Concepts, San Antonio, TX, US) was applied to the wound and IV antibiotics were continued.

**Figure 3:**
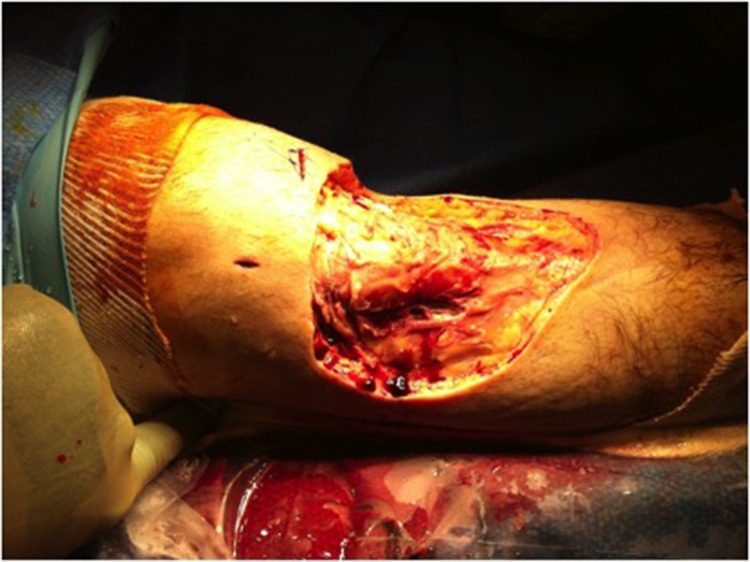
Wound following extensive surgical debridement over a 12-day period

Over the next 12 days, the wound failed to settle and a further 5 surgical debridements were performed. A large area of skin ulceration was evident over the anteromedial aspect of the right proximal tibia, measuring approximately 15cm × 15cm (Fig 3). Despite sending multiple microbiology specimens at each stage, we failed to grow any bacterial organism. A dermatology opinion was gained, and a diagnosis of PG was made on the basis of the clinical findings and the histology results taken from a skin biopsy. The patient was commenced immediately on high dose oral steroid therapy. Sixteen weeks after his primary ACL reconstruction, the wound had fully healed with no requirement for plastic surgical intervention or skin grafting. The ACL graft remains intact.

## Discussion

PG is one of the most dramatic and rapidly progressing skin conditions. It was first reported by Brunsting *et al* in 1930 when they published a series of five cases presenting with inflammatory sterile neutrophilic dermatosis.^1^ The cases were misdiagnosed initially as infection. Since then, PG has been a diagnostic dilemma for clinicians. Due to the unknown aetiology, there is no clear diagnostic test; microbiology cultures are negative and histopathology is usually not definitive.

PG can be idiopathic or associated with other conditions such as inflammatory bowel disease, myeloproliferative disease, Wegener’s granulomatosis and, more worryingly, it can be paraneoplastic,^2^ requiring rapid diagnosis and appropriate treatment. It can also present after surgery, especially abdominal and breast surgery. To our knowledge, only six orthopaedic cases have been reported with PG following surgery. These cases occurred after hip and knee arthroplasty, knee arthroscopy, tarsal tunnel release and an operation on a rheumatoid hand.^3,4^

The patients present with a rapidly developing pustule surrounded by an area of erythema and oedema. It then ulcerates, developing undermined borders with bluish colouration. These ulcers can develop a purulent cover. They can become painful and the patient develops systemic symptoms. Blood tests show raised inflammatory markers. Histopathology is not specific; it shows leucocytoclastic vasculitis and sometimes necrotising granulomatous inflammatory changes.^5^ These features are often misdiagnosed as infection. While PG is treated by immunosuppressive therapy, post-operative wound infections are managed with high dose IV antibiotics and surgical debridement as these can be limb or even life threatening.

All the reported cases, including our patient, had high doses of IV antibiotics and underwent multiple surgical debridement and lavage procedures. This often resulted in the development of skin and soft tissue defects that required a prolonged hospital stay and plastic surgical intervention. In some cases, the decision to amputate or perform an arthrodesis of the limb was considered.^3,4^

In the present case, the surgical wound did not respond to standard surgical management or IV antibiotic therapy. A dermatology opinion was therefore sought and the diagnosis of PG was made. The patient responded to systemic corticosteroid treatment and did not require any plastic surgical input.

## Conclusions

We believe PG must be considered in all cases of suspected wound infection that are refractory to standard treatment options. PG is often a clinical diagnosis of exclusion and an early opinion from an experienced dermatologist should be sought in these cases with samples sent for histology along with the microbiology samples, especially in cases at risk or those not responding to appropriate antibiotics and surgical treatment.
